# Development and psychometric analyses of a mentee competency self-assessment (MCSA) tool

**DOI:** 10.1017/cts.2026.10727

**Published:** 2026-03-25

**Authors:** So Hee Hyun, Jonathan M. Orsini, Kimberly Spencer, Stephanie C. House, Christine Pfund

**Affiliations:** 1 Institute for Clinical and Translational Research, University of Wisconsin-Madison, USA; 2 Office of the Provost, University of Florida, USA; 3 Wisconsin Center for Education Research, University of Wisconsin-Madison, USA

**Keywords:** *Mentoring Up* workshop, mentee competency self-assessment, mentorship instrument, mentorship skills, mentorship common measures

## Abstract

**Introduction:**

This paper presents the development and psychometric analyses of the Mentee Competency Self-Assessment (MCSA), a tool designed to evaluate mentee skills in research mentoring relationships and assess the *Mentoring Up* curriculum. By assessing mentee competencies across diverse settings, the study aims to enhance understanding of mentoring dynamics and highlight the importance of considering both mentor and mentee perspectives in assessing mentorship effectiveness.

**Methods:**

The 26-item MCSA instrument was developed based on the validated Mentoring Competency Assessment (MCA) to evaluate the *Mentoring Up* curriculum. Data was obtained from 401 mentees who attended *Mentoring Up* workshops between 2015 and 2022. Principal component analysis (PCA) was performed with varimax rotation to examine the internal structure of the MCSA. A team of mentoring experts independently reviewed and reached consensus on component alignment. Confirmatory factor analysis (CFA) and internal consistency were performed to assess construct validity and reliability.

**Results:**

Factor and reliability analyses support an eight-component structure of a 22-item MCSA. All parameter estimates were significant, and the components demonstrated acceptable to high internal consistency (Cronbach’s alpha = 0.58–0.90).

**Conclusions:**

The final 22-item scale (MCSA-22) aligns with eight competencies and is now suited for measuring mentee mentorship skills. Given the modest sample size and other study limitations, replication of this proposed modification of the MCSA is an important next step. Additional recommendations for future scale development are offered.

## Introduction

Effective research mentoring relationships are important [1–4]. As noted in the 2019 NASEM report on The Science of Effective Mentorship, “Mentorship is a professional, working alliance in which individuals work together over time to support the personal and professional growth, development, and success of the relational partners through the provision of career and psychosocial support [1].” In this working alliance, it is essential to understand and support the roles, skills, and agency of both mentees and mentors. Thus, there has been an emphasis on empowering mentees to be proactive and develop the knowledge, skills, and confidence they need to navigate their mentoring relationships effectively [5–7].

Mentorship education programs, especially those using evidence-based approaches, can support the development of skills for mentors and mentees to work effectively in their mentoring relationships. One evidence-based approach to mentor training is the well-studied *Entering Mentoring* curriculum [8,9], which focuses on competencies such as Maintaining Effective Communication, Aligning Expectations, Promoting Professional Development, among others. To address the bi-directional nature of the mentoring relationship, a complementary companion curriculum, *Mentoring Up*, was first implemented in 2015 to support junior faculty and postdoctoral fellows to be more proactive in their mentorship relationships and the development of their own careers [6,7]. The *Mentoring Up* competencies mirror those in *Entering Mentoring* but focus on the role of the mentee in a two-way collaborative relationship. For example, the *Mentoring Up* module Seeking Professional Development is used instead of the *Entering Mentoring* module, Promoting Professional Development.

Assessment of both curricula is necessary to understand training impact and outcomes. The Mentoring Competency Assessment (MCA) assessment tool was originally designed to evaluate the effectiveness of an *Entering Mentoring*-based curriculum adaptation through the assessment of mentors’ skills [10]. The 26 items in the original MCA were aligned with the curriculum’s learning objectives across six competencies [11]. Two versions of the tool were created, one for the mentors to assess themselves, and the other for mentees to assess their mentors [12]. Both original versions of the tool were validated for construct validity and reliability using data from mentors and mentees in academic health settings. The mentor version was later revalidated through confirmatory factor analysis (CFA) and internal consistency testing with a more diverse population of mentors [13]. The MCA has further been used in its original or adapted form across diverse contexts to assess mentors’ skills [14–19].

Despite the growing emphasis of the mentee’s role in the mentoring relationship, few tools are available to specifically assess the mentorship skills and competencies of the mentee [20]. Many existing tools focus on mentoring program outcomes or mentor effectiveness, either through mentor self-assessment or the mentee’s assessment of the mentor [21–24]. Additionally, tools developed to assess mentee skills, such as the Entering Research Learning Assessment or Undergraduate Research Student Self-Assessment, often focus primarily on research skills or program outcomes and not mentoring relationship skills specifically [25,26].

To assess the *Mentoring Up* curriculum, a new tool was developed, the Mentee Competency Self-Assessment (MCSA). The MCSA has been used to evaluate the impact of mentorship training on mentee outcomes, including perceived gains in skills such as maintaining effective communication or aligning expectations following participation in the *Mentoring Up* workshop [27]. Here, we report on the development and psychometric analyses of the MCSA tool to assess the skill gains of mentees who participated in *Mentoring Up* events using a large, broad population of mentees across different settings and contexts.

## Methods

### Respondents and procedure

*Mentoring Up* training evaluation data, which includes data from the new MCSA, were collected nationally from 2015 to 2022. The original dataset included survey responses from 911 mentees who collectively attended 70 *Mentoring Up* training events hosted by 35 institutions or organizations across the United States and Puerto Rico. Eleven training events incorporated only a single competency module or had no participant information and were excluded from analysis, accounting for the exclusion of 510 mentees. The final dataset includes survey responses from 401 mentees who attended 59 *Mentoring Up* in-person or online events hosted by 34 institutions/organizations and consented to having their data used for research. Across institutions, 15 were public, 13 private, 9 land-grant, 19 either R1 or R2, and 6 Hispanic-serving colleges or universities. There were no institutions categorized as liberal arts or historically black colleges or universities. Participant data from events associated with 7 professional organizations or societies were also included. Eight of the institutions or organizations held repeat events, ranging from 2 to 13 events.

Table 1 shows sample characteristics of the respondents on which the psychometric properties of the measure were tested in this study. For gender distribution, 36% (*n* = 143) of mentees identified as male, 61% (*n* = 245) as female, and 2% (*n* = 7) identified as “other,” including Transgender, Intersex, Nonbinary, etc. In terms of race and ethnicity, the majority were non-Hispanic/Latino (86%, *n* = 336) and 56% (*n* = 223) self-identified as white. The next largest groups were Asian (22%, *n* = 87), Hispanic (13%, *n* = 51), and Black/African American (13%, *n* = 48). Most respondents were graduate students (40%, *n* = 160) or postdoctoral fellows (31%, *n* = 123). Only 33% (*n* = 89) reported having participated in a prior mentorship training workshop.

**Table 1.** Characteristics of respondents

Characteristics	Mentees (*N* = 401)
Gender*, no. (%)	
Female	245 (61.1)
Male	143 (35.7)
Other (transgender, intersex, nonbinary, etc.)	7 (1.7)
Prefer not to report	4 (1.0)
Ethnicity*, no. (%)	
Non-Hispanic or Latino	336 (85.5)
Hispanic or Latino	51 (12.9)
Prefer not to report	6 (1.5)
Race*, no. (%)	
American Indian or Alaskan Native	6 (1.5)
Native Hawaiian or Pacific Islander	1 (0.2)
Asian	87 (21.7)
Black or African American	48 (12.6)
White	223 (55.6)
Other	15 (3.7)
Prefer not to report	11 (2.7)
Academic rank*, no. (%)	
Early career faculty	73 (18.2)
Late career faculty	4 (1.0)
Research staff	42 (10.4)
Graduate student	160 (39.9)
Postdoctoral fellow	123 (30.7)
Undergraduate student	10 (2.5)
Other (Lecturer, Clinical fellow, Medical students, etc.)	39 (9.6)
**Prior mentorship training workshop participation, no. (%)**	
Yes	89 (32.6)
No	184 (67.4)

* *Note:* Participants were allowed to select multiple responses; therefore, the reported *n* and percentages reflect the number of selections rather than unique participants.

### Instrument

A new self-assessment tool, the MCSA, was used in this study to assess the effectiveness of the *Mentoring Up* curriculum from the mentee perspective. The MCSA was derived from the validated Mentoring Competency Assessment (MCA), which was originally developed to assess mentor skill gains [11].

Using the original MCA tool as a base, each scale item was reviewed individually and revised to align with the *Mentoring Up* curriculum and the role of the mentee [7,28]. In some cases, no changes were made to the item because the phrasing was role-inclusive (e.g., active listening). In other cases, edits were incorporated to acknowledge the specific role of the mentee, such as changing the MCA item, “working with mentees to set clear expectations of the mentoring relationship” to read, “working with your primary mentor to set clear expectations of the mentoring relationship” in the MCSA. Likewise, the MCA item “providing constructive feedback,” was revised to “receiving constructive feedback.” During the review process, no new scale items were introduced or removed from the final set of items. The adjusted MCSA instrument consisted of 26 items on a Likert-type scale (1 = Not at all skilled, 4 = Moderately skilled, 7 = Extremely skilled).

Mentees self-reported their skill levels on a retrospective pre- and post-assessment across the 26 items, which were organized into six subscales paralleling the subscales of the MCA. The pre- and post-scores were collected simultaneously, immediately following completion of the workshop. The post-scores were used in the psychometric analysis. The original subscales include: (a) Maintaining Effective Communication (6 items), (b) Aligning Expectations (5 items), (c) Assessing Understanding (3 items), (d) Fostering Independence (5 items), (e) Addressing Diversity (2 items), and (f) Seeking Professional Development (5 items).

### Analysis

To examine the internal structure of the MCSA, Principal Component Analysis (PCA) with varimax rotation was used. PCA is a multivariate technique typically used to cut a large set of variables to a small set, while containing as much of the information (variation) as possible. The criteria used were based primarily on Hatcher’s guidelines [29], with additional considerations from other sources [30–33]. The following criteria were applied:

1. Scree plot: The point of inflection in the scree plot was used to help determine the number of components to retain, consistent with Hatcher’s recommendations.
2. Eigenvalue criterion: Hatcher’s criterion typically uses an eigenvalue cutoff of 1.0. Additional literature suggests that more flexibility may be needed in some cases [30–32]. In this study, a slightly more liberal cutoff of 0.75 was used to decide how many components to retain and interpret. This cutoff was chosen to balance extracting meaningful factors while ensuring that the retained components explain a sufficient portion of the variance, particularly in smaller datasets. We acknowledge that the use of 0.75 as a cutoff slightly deviates from the conventional eigenvalue-one criterion, but it was adopted because the MCSA items were directly derived from the validated MCA items, with modest language changes, making it important to reflect the most meaningful items in the analysis [11,12].
3. Proportion of variance explained: Analysis required retained components account for at least 50% of the total variance consistent with Hatcher’s guidelines.
4. Factor loadings: A component was considered meaningful if its variables had significant loadings, with a cutoff point of 0.30 for factor loadings. This threshold is consistent with common practice and serves as an additional criterion not explicitly mentioned by Hatcher, but commonly used in factor analysis studies [30,33].

After identifying the number of meaningful components to include for the MCSA, a team of mentorship experts (the authors and JR) independently interpreted the factors for all relevant results, discussed their interpretations, and reached consensus on the interpretations of the factors and their alignment. Finally, CFA was conducted using the eight components retained from the PCA as the hypothesized structure. There was no prespecified, theory-driven hypothesis about which items would align with which components prior to the CFA, as the MCSA was derived from the validated MCA measure [11,12]. Instead, the CFA served to confirm the structure revealed in the exploratory analysis [34]. Maximum likelihood estimation was used to evaluate model fit, and internal consistency reliability was assessed using Cronbach’s alpha. Although the CFA and PCA were conducted on the same sample, potentially limiting independence, the analyses provide meaningful evidence of construct validity and reliability for the MCSA. The data were analyzed using Stata SE 16.0 for Mac (Stata Corp, College Station, Texas).

## Results

### Principal component analysis

**Figure 1.**
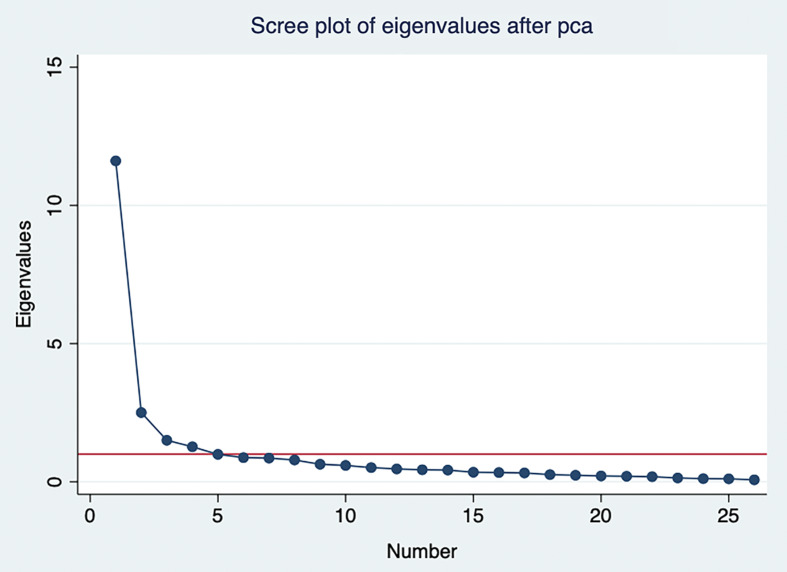
Scree plot of the eigenvalues of the factors. The scree plot displays the eigenvalues (*y*-axis) of the factors or principal components (*x*-axis) extracted from the dataset. Each point on the plot represents the eigenvalue of a specific factor or component. The plot is used to visualize the relative importance of each factor, with the eigenvalues typically decreasing as the number of factors increases. The “elbow” of the plot, where the rate of decrease sharply changes, indicates the optimal number of factors to retain for further analysis. In this plot, factors with eigenvalues greater than 1 are generally considered significant.

A scree plot showed a sharp point of inflection after the first component (Figure 1). Only four components had initial eigenvalues >1, with values ranging from 1.27 to 11.61. While the 26-item MCSA was originally developed with six subscales, this study applied a cutoff on the eigenvalues of 0.75 to determine how many components should be retained to explain most items of the MCSA. Considering the eigenvalue and the “proportion of variance accounted for” criterion, the first 8 components were taken as the starting point for analysis which explained most items of the MCSA with 79% of variation.

Table 2 shows significant component loadings of the 8 components with varimax rotation; 22 of the total 26 items were loaded into those components. First, items of the maintaining effective communication subscale and items of the fostering independence subscale were mixed and loaded into three different components. The three items, (3) establishing a relationship based on trust, (4) identifying and accommodating different communication styles and (19) negotiating a path to professional independence with your primary mentor, were loaded into one component. Two items, (1) active listening and (2) receiving constructive feedback, were loaded into another component, while the items of (5) employing strategies to improve communication with your primary mentor and (6) helping your primary mentor coordinate with your other mentors, were not loaded into any components. Four items, (15) motivating yourself, (16) building your confidence, (17) stimulating your creativity, and (18) acknowledging your professional contributions, were loaded into a separate component.

**Table 2.** Item loadings of the 8 components with varimax rotation (Blank if a loading <0.30)

No.	Item	Component							
		1	2	3	4	5	6	7	8
**Maintaining effective communication**									
1	Active listening	–	–	–	–	–	–	0.61	–
2	Receiving constructive feedback	–	–	–	–	–	–	0.64	–
3	Establishing a relationship based on trust	–	–	–	0.48	–	–	–	–
4	Identifying and accommodating different communication styles	–	–	–	0.50	–	–	–	–
5	Employing strategies to improve communication with your primary mentor	–	–	–	–	–	–	–	–
6	Helping your primary mentor coordinate effectively with your other mentors	–	–	–	–	–	–	–	–
**Aligning Expectations**									
7	Working with your primary mentor to set clear expectations of the mentoring relationship	–	0.54	–	–	–	–	–	–
8	Aligning your expectations with your primary mentor's	–	0.54	–	–	–	–	–	–
9	Considering how personal and professional differences may impact expectations	–	–	–	–	–	0.35	–	–
10	Working with your primary mentor to set research goals	–	0.39	–	–	–	–	–	–
11	Developing strategies to meet goals	–	–	–	–	–	–	–	–
**Assessing understanding**									
12	Accurately estimating your level of scientific knowledge	0.46	–	–	–	–	–	–	–
13	Accurately estimating your ability to conduct research	0.54	–	–	–	–	–	–	–
14	Employing strategies to enhance your knowledge and abilities	0.43	–	–	–	–	–	–	–
**Fostering independence**		–	–	–	–	–	–	–	–
15	Motivating yourself	–	–	0.35	–	–	–	–	–
16	Building your confidence	–	–	0.53	–	–	–	–	–
17	Stimulating your creativity	–	–	0.39	–	–	–	–	–
18	Acknowledging your professional contributions	–	–	0.45	–	–	–	–	–
19	Negotiating a path to professional independence with your primary mentor	–	–	–	0.44	–	–	–	–
**Addressing diversity**		–	–	–	–	–	–	–	–
20	Taking into account the biases and prejudices you bring to the mentor/mentee relationship	–	–	–	–	–	0.50	–	–
21	Working effectively with mentors whose personal background is different from your own (age, race, gender, class, region, culture, religion, family composition etc.)	–	–	–	–	–	0.60	–	–
**Seeking professional development**		–	–	–	–	–	–	–	–
22	Networking effectively	–	–	–	–	0.67	–	–	–
23	Setting career goals	–	–	–	–	–	–	–	–
24	Balancing work with your personal life	–	–	–	–	–	–	–	0.65
25	Understanding your impact as role model	–	–	–	–	–	–	–	0.54
26	Acquiring resources (e.g., grants, etc.)	–	–	–	–	0.52	–	–	–

Second, in measuring the MCSA assessing understanding subscale, three items significantly loaded into one component, (12) accurately estimating your level of scientific knowledge, (13) accurately estimating your ability to conduct research and (14) enhancing knowledge and abilities.

Third, items from the aligning expectations and addressing diversity subscales were mixed and loaded into two different components. Three items, (7) working with your primary mentor to set clear expectations of the mentoring relationship, (8) aligning your expectations with your primary mentor’s and (10) working with your primary mentor to set research goals, were loaded into one component, while item 11, developing strategies to meet goals, was not loaded into any components. Three items, (9) considering how personal and professional differences may impact expectations, (20) taking into account the biases and prejudices you bring to the mentor/mentee relationship and (21) working effectively with mentors whose personal background is different from your own, were loaded into a single component.

Lastly, five items from the seeking professional development scale were split into two different components. The two items, (22) networking effectively and (26) acquiring resources, were loaded into one component. Two other items, (24) balancing work with your personal life and (25) understanding your impact as a role model, were loaded into the separate component. Item 23, setting career goals, was not loaded into any components.

### Confirmatory factor analysis and reliability analysis

To assess the construct validity and reliability of the MCSA with its eight components, CFA and Cronbach’s alpha were conducted. Based on the results of the PCA, which identified eight components from the 22-item MCSA, the CFA was used to confirm the structure revealed by the PCA, rather than to test a separate, a priori hypothesis about item-to-component alignments. Maximum likelihood estimation was applied to evaluate how well the items measured the eight identified components, and model fit was assessed using several goodness-of-fit indices: chi-square, root mean square error of approximation (RMSEA), comparative fit index (CFI), Tucker–Lewis index (TLI), and standardized root mean square residual (SRMR).

Four items, (5) employing strategies to improve communication with your primary mentor, (6) helping your primary mentor coordinate with your other mentors, (11) developing strategies to meet goals, and (23) setting career goals, were excluded from the factor analysis and Cronbach’s alpha analysis, as they were not loaded into any components in the PCA.

**Figure 2.**
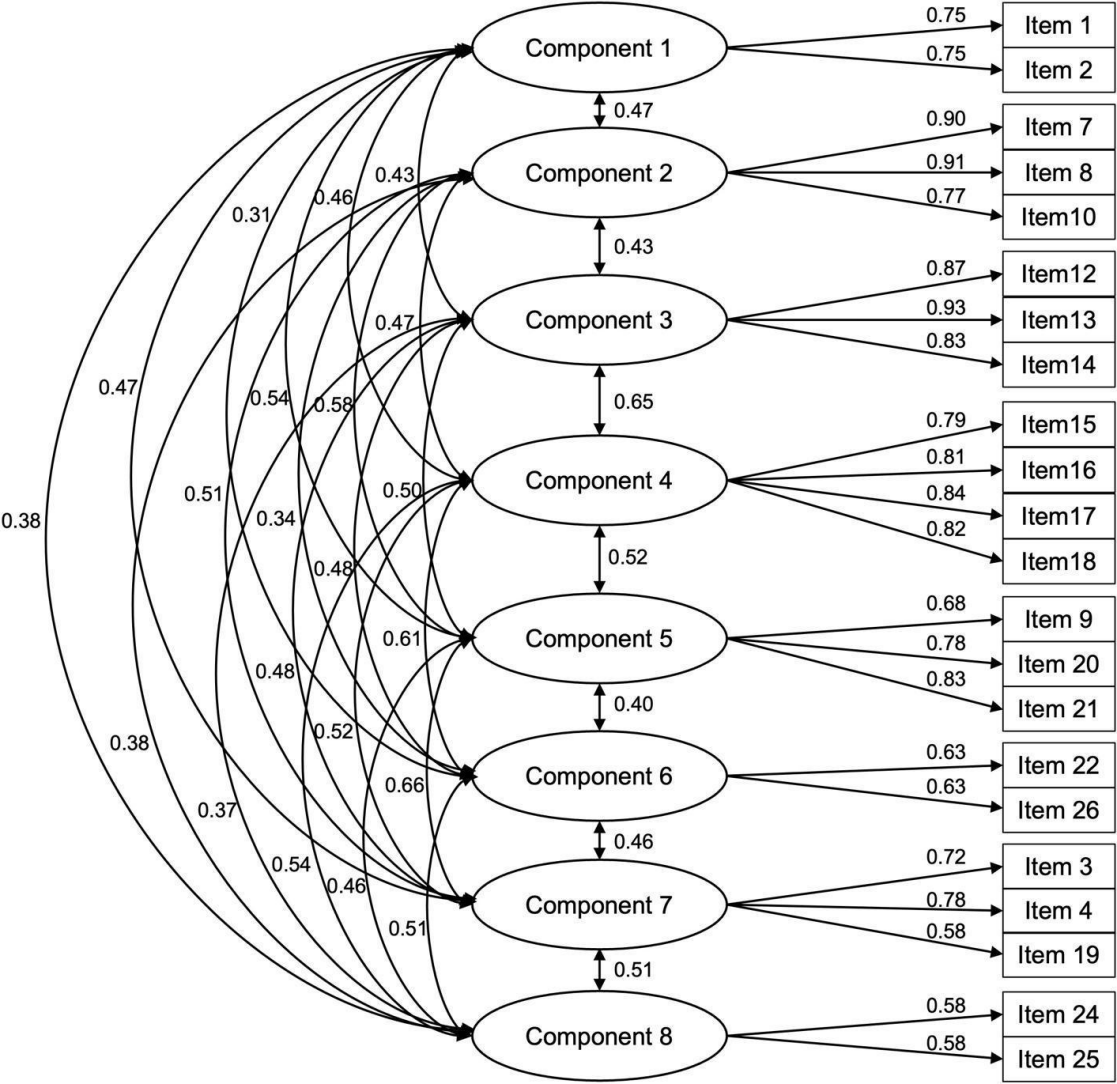
Confirmatory factor analysis (CFA) model with eight latent components. This figure presents the CFA model estimating eight latent components representing distinct conceptual domains. Circles indicate latent components, and rectangles represent observed items. Single-headed arrows show factor loadings from each component to its items; double-headed arrows represent estimated correlations between latent components. All paths shown are statistically significant at *p* < 0.05 and standardized estimates are reported.

Table 3 shows standardized factor loadings and Cronbach’s alpha scores for the eight components of the 22-item MCSA. The following indices were described and measured in combination: chi-square; RMSEA; CFI; TLI; and SRMR [35]. A eight-component structure was validated (*χ* ^2^ = 313.209, *p* < 0.001, RMSEA = 0.083, CFI = 0.907, TLI = 0.881, SRMR = 0.073) and the hypothesized model of the eight components resulted in an acceptable fit to the data: RMSEA > 0.08; CFI > 0.80; TLI > 0.80; and SRMR > 0.05. Considering the sensitivity of the chi-square statistic to sample size, overall goodness-of-fit indices were used to determine model adequacy. All the parameter estimates for each item were significant, with standardized factor loadings ranging from 0.58 to 0.93. The alpha coefficient for the eight components is from 0.58 to 0.90, suggesting that the items have relatively high internal consistency. Figure 2 illustrates the CFA model with the eight latent components and their corresponding items.

**Table 3.** Factor loadings and Cronbach's alpha scores for the 22-item MCSA

No.	Item	Factor loading	Cronbach’s alpha
**Component 1: maintaining effective communication**			
1	Active listening	0.75	0.75
2	Receiving constructive feedback	0.75	
**Component 2: aligning expectations**			
7	Working with your primary mentor to set clear expectations of the mentoring relationship	0.90	0.90
8	Aligning your expectations with your primary mentor’s	0.91	
10	Working with your primary mentor to set research goals	0.77	
**Component 3: assessing understanding**			
12	Accurately estimating your level of scientific knowledge	0.87	0.90
13	Accurately estimating your ability to conduct research	0.93	
14	Employing strategies to enhance your knowledge and abilities	0.83	
**Component 4: fostering growth/confidence**			
15	Motivating yourself	0.79	0.88
16	Building your confidence	0.81	
17	Stimulating your creativity	0.84	
18	Acknowledging your professional contributions	0.82	
**Component 5: addressing diversity**			
9	Considering how personal and professional differences may impact expectations	0.68	0.77
20	Taking into account the biases and prejudices you bring to the mentor/mentee relationship	0.78	
21	Working effectively with mentors whose personal background is different from your own (age, race, gender, class, region, culture, religion, family composition etc.)	0.83	
**Component 6: seeking professional development**			
22	Networking effectively	0.63	0.65
26	Acquiring resources (e.g., grants, etc.)	0.63	
**Component 7: building trust**			
3	Establishing a relationship based on trust	0.72	0.74
4	Identifying and accommodating different communication styles	0.78	
19	Negotiating a path to professional independence with your primary mentor	0.58	
**Component 8: enhancing work–life integration**			
24	Balancing work with your personal life	0.58	0.58
25	Understanding your impact as role model	0.58	

## Discussion

The MCSA was developed to evaluate interventions designed to help mentees take a more proactive role in their mentoring relationships. Here we report on the development and psychometric analyses of the MCSA tool to assess skill gains of a broad population of mentees who participated in *Mentoring Up* training events. The results provide preliminary evidence supporting the MCSA as a promising assessment approach. Results also suggest differences from its MCA parent instrument designed to assess mentor skills [11,12]. The revised tool, MCSA-22 has eight principal components, each of which is discussed below, with comparison to the MCA.

### Maintaining effective communication: building clarity and empathy

Effective communication remained a central component, emphasizing active listening, feedback exchange, and expressing empathy, while trust-building and accommodating communication styles formed a separate component, which we named “building trust [36].” This change from the original MCA suggests that mentees at early career stages may not yet perceive interpersonal trust as an integral part of effective communication [37].

### Aligning expectations: establishing shared goals and understanding

The aligning expectations component clustered items on setting expectations and setting research goals which had not previously loaded together in the MCA. This result highlights a clear distinction between “goals” and “expectations” seen by mentors, but perhaps not mentees. Expectations are beliefs about how one behaves, or should behave, while goals are something one must plan for and work towards. This distinction suggests that mentees may conceptualize goals and expectations differently, especially when connected to a specific mentoring relationship. The items that referenced working directly with a primary mentor clustered together, whereas items with more abstract language loaded differently. For example, the item “developing strategies to meet goals” did not load at all and the item, “considering how personal and professional differences may impact expectations” loaded with the addressing diversity component. Because mentees may be focused on achieving specific goals related to their career or research, they may have a narrower interpretation of goals and expectations.

### Assessing understanding: developing self-awareness of knowledge and skills

This component retained the same structure as the original MCA, encompassing items related to mentees’ self-evaluation of their scientific knowledge and research skills, including strategies to enhance these abilities [12].

### Fostering growth and confidence: strengthening self-efficacy and motivation

This component emphasized mentees’ perceptions of motivation, confidence, creativity, and professional recognition, which are all tied to feelings of competence, either as experienced internally or reinforced externally. The item, negotiating a path to professional independence, which had been part of the same construct in the original MCA, here loaded separately with building trust. Mentees appear to assess their comfort level operating independently from the relational processes needed to act more independently.

### Addressing diversity: fostering awareness and inclusion

Items in this component retained their original structure, grouping recognizing personal biases, working with diverse mentors, and considering how differences affect expectations. These items aligned with previous MCA results [12].

### Seeking professional development: acquiring resources and networks

This component focused on mentees’ proactive pursuit of growth opportunities, such as networking and acquiring resources for professional development. Items that loaded with these in the original MCA, balancing work and personal life and being a role model, loaded into a new component (Enhancing Work–Life Integration, discussed below). Setting career goals did not load, possibly because mentees view it as an implicit part of expectation alignment, rather than something they seek for their own development.

### Building trust: creating a foundation for independence

This newly emergent component clustered trust-building, accommodating communication styles, and negotiating independence. This new component may reflect the importance of psychological safety in building effective interpersonal relationships, especially those that involve power differentials [38]. Research has shown that building trust, sharing knowledge, being oneself, and speaking up are associated with psychological safety. Past research has also shown that building trust requires benevolence, integrity, predictability, and competence, which could also be seen as antecedents for accommodating the communication styles of others, and negotiating independence [39,40].

### Enhancing work-life integration: modeling balance and well-being

This component coalesced as a distinct component, grouping work–life balance and understanding one’s impact as a role model. Literature on the importance of mentors being role models supports the mentee perspective that these should load together [41,42]. Graduate students often connect these two behaviors in how they assess effective mentorship, emphasizing that the quality of the mentor and the environment they create, including the norms they model, can be as important as professional development outcomes [43].

### Mentee and mentor perspectives: distinct but complementary

Several key differences emerged when comparing the MCSA-22 to previous MCA versions, indicating that mentee and mentor assessments may diverge in structure and perception. First, items like improving communication (item 5) and understanding one’s impact as a role model (item 25) failed to load when the MCA was revalidated, but item 25 loaded in this study, possibly reflecting its relevance to mentees [44–46]. Second, trust-related items diverged from the communication component, which may reflect differing perceptions of power dynamics between mentors and mentees. Third, items on self-efficacy and independence, split into separate components in the MCA, loaded together here, suggesting mentees view them as a unified construct. Finally, wording like “employing strategies” (items 5 and 11) may have confused participants, potentially affecting item performance.

There are several factors that may contribute to these differences. First, the mentee sample was earlier in career stage, with 74.1% students or fellows versus more faculty-heavy samples in past MCA studies [12,14]. Second, the *Mentoring Up* curriculum that the mentees engaged with was comprised of different activities and content than its companion mentor version, which may have contributed to different learning outcomes. Third, the current analysis of the MCSA-22 was based on mentees’ self-assessments of their own skills, whereas the MCA was developed using both mentor self-ratings and mentee ratings of their mentors [11,12]. This distinction likely influences how the items were interpreted.

Despite many differences, significant areas of similarity between the current analysis of the MCSA-22 and the past MCA revalidation included Assessing Understanding and Addressing Diversity [12]. The same items loaded together in both studies, suggesting that they are perceived similarly by both mentors and mentees, reflecting shared recognition of key mentorship competencies.

Overall, these findings highlight the importance of developing and using mentee-specific tools that capture the unique perspectives and competencies of mentees in mentoring relationships. The distinct factor structure of the MCSA-22 highlights that mentees experience and interpret mentorship skills differently than mentors, reinforcing the need for tailored assessment and training approaches. Future research should continue to refine and test the MCSA-22, explore its application across diverse populations, and examine how mentee competencies evolve over time. By better understanding and supporting mentees’ roles, mentorship programs can more effectively foster productive, reciprocal mentoring relationships that promote career development and personal growth.

## Study limitations

Although this study provides valuable insights into measures of mentees’ mentorship skills, there are several limitations that should be considered:

1. Pilot testing and cognitive interviewing: Using the original MCA, items were adjusted for the MCSA without additional piloting or cognitive interviewing to ensure participants understood the wording of the items (response process) or to assess how the adapted MCSA items were interpreted. Future studies should consider piloting revised instruments to ensure clarity and proper interpretation by the target audience.
2. Sample size, diversity, and self-reported data: This study involved a relatively small sample size of mentees (*n* = 401), which was significantly smaller and less demographically diverse compared to the mentor sample (*n* = 1759) used in revalidating the MCA measure for mentors [12]. The data for this study were collected through self-reported surveys which are prone to biases such as social desirability and recall errors thus potentially impacting the accuracy and reliability of our results. However, we acknowledge that self-reported data is commonly used in studies of mentoring programs and mentoring relationships, with established literature supporting its value in this context [47].
3. Incomplete representation of *Mentoring Up* curriculum: The survey instrument used in this study was adapted from the MCA for mentors, and it did not fully reflect all components of the *Mentoring Up* curriculum. Notably, self-efficacy, an important competency, was not included in the scale, suggesting that the measure might not comprehensively capture all aspects of mentees’ mentorship skills.
4. Potential overfitting in PCA and CFA analyses: Both PCA and CFA were conducted on the same sample, potentially resulting in overfitting. While the study established a strong argument for the psychometric properties of the scale, it is possible that the model does not fully represent the latent constructs. The items used in the model were based on a set of a priori concepts developed from the mentor’s perspective, which likely missed some components of the latent variable. While CFA was used to confirm the eight-component structure identified in the PCA, there was no explicit a priori hypothesis about which items would align with which components, as the MCSA items were derived from the validated MCA measure. As a result, the CFA primarily served to confirm patterns emerging from the PCA rather than testing a separate, distinct hypothesis. This approach provides useful evidence of construct validity but limits the extent to which the CFA can be considered a fully confirmatory test.

To address these methodological limitations, future studies should prioritize the development and validation of mentee-specific assessment tools through iterative processes, including piloting, cognitive interviewing, and engagement with mentees in the design phase and analyses phases. Expanding sample sizes and enhancing participant diversity will be critical for improving the generalizability of findings. In addition, incorporating multiple data sources such as mentor assessments or observational data could help triangulate self-reported responses. Finally, future psychometric analyses should be conducted on independent samples or with cross-validation techniques to reduce the risk of overfitting and strengthen confidence in the scale’s structure and utility.

## Future directions and recommendations

The 22-item MCSA-22 offers a promising albeit preliminary approach for evaluating a subset of mentee mentorship skills. Further replication and refinement are necessary before the measure can be recommended for widespread or high-stakes use in practice. The results presented here indicate the tool is ready for use in the evaluation of mentee training interventions and as a basis for futureinstrument development and testing with more extensive research. At this stage, the MCSA-22 is suited for developmental, formative, and research purposes, where results are interpreted with appropriate caution.

Further testing of the revised instrument would ensure clarity and accurate interpretation. Such research should include the perspectives of mentees to better understand how to interpret current factor loadings. Cognitive interviewing could also be done on a revised scale to assess researcher and mentee interpretations.

Future research might also examine studies of mentee competencies across career stages and contexts. We note that postdoctoral fellows and graduate students are at different stages of their educational journeys. Previous research has shown that transition points are important in understanding how nascent scholars integrate into competitive and sometime socially isolating academic environments [48]. Given that, future research should analyze the MCSA-22 using distinct populations, separating graduate students and postdocs, to see if the results are as different as between the current study and the past revalidation using mentors [12]. Additionally, exploration of mentees in various contexts (disciplines, institution type, etc.) would be useful. This additional research would test the validity of the MCSA outside of the context of mentee training evaluation.

## Conclusion

This study investigated the psychometric properties of the MCSA, a developing tool designed to assess mentee skills in research mentoring relationships, specifically aligned with the *Mentoring Up* curriculum [6,7]. The results suggest that the structure of the MCSA differs from its mentor-specific predecessor (the MCA). Although the 26-item MCSA was initially tested with six subscales, factor and reliability analyses ultimately recommended a streamlined 22-item scale with eight components for measuring mentee mentorship skills. (The full list of MCSA-22 items is provided in Supplementary Table 1). These results provide preliminary support for the MCSA-22 as a promising approach for assessing mentee training, supporting mentees in self-evaluating areas for growth, and assisting mentors in identifying ways to better support mentee development. Additional validation with independent samples is needed before broader application is recommended.

## Supporting information

10.1017/cts.2026.10727.sm001 Hyun et al. supplementary material Hyun et al. supplementary material

## Data Availability

None.

## Supplementary material

The supplementary material for this article can be found at https://doi.org/10.1017/cts.2026.10727.

## Previous presentations

Poster presentation at the Translational Science 2024 conference in Las Vegas, NV on April 4, 2024.
