# Supplementary material for: Development and psychometric analyses of a mentee competency self-assessment (MCSA) tool
Source: J Clin Transl Sci. 2026 Mar 25;10(1):e70. doi: 10.1017/cts.2026.10727 (PMC13107358; doi:10.1017/cts.2026.10727)
Supplement: Hyun et al. supplementary material [file S2059866126107274sup001.docx]

**Supplementary table 1.** Mentee Competency Self-Assessment (MCSA)-22 (Based on 22 items)^^[[1]](#footnote-1)^^

| **No.** | **Item^^[[2]](#footnote-2)^^** |
| --- | --- |
| **Maintaining Effective Communication** | |
| 1 | Active listening |
| 2 | Receiving constructive feedback |
| **Aligning Expectations** | |
| 3 | Working with your primary mentor to set clear expectations of the mentoring relationship |
| 4 | Aligning your expectations with your primary mentor's |
| 5 | Working with your primary mentor to set research goals |
| **Assessing Understanding** | |
| 6 | Accurately estimating your level of scientific knowledge |
| 7 | Accurately estimating your ability to conduct research |
| 8 | Employing strategies to enhance your knowledge and abilities |
| **Fostering Growth/Confidence** | |
| 9 | Motivating yourself |
| 10 | Building your confidence |
| 11 | Stimulating your creativity |
| 12 | Acknowledging your professional contributions |
| **Addressing Diversity** | |
| 13 | Considering how personal and professional differences may impact expectations |
| 14 | Taking into account the biases and prejudices you bring to the mentor/mentee relationship |
| 15 | Working effectively with mentors whose personal background is different from your own (age, race, gender, class, region, culture, religion, family composition etc.) |
| **Seeking Professional Development** | |
| 16 | Networking effectively |
| 17 | Acquiring resources (e.g. grants, etc.) |
| **Building Trust** | |
| 18 | Establishing a relationship based on trust |
| 19 | Identifying and accommodating different communication styles |
| 20 | Negotiating a path to professional independence with your primary mentor |
| **Enhancing Work-Life Integration** | |
| 21 | Balancing work with your personal life |
| 22 | Understanding your impact as role model |

1. Question prompt: Please rate how skilled you feel you were BEFORE the workshop and how skilled you feel you are NOW in each of the following areas. How skilled were/are you in … [↑](#footnote-ref-1)
2. Using seven-point Likert scale (1=not at all skilled, 2, 3, 4=moderately skilled, 5, 6, 7=extremely skilled) and asking mentors to rate a retrospective pre score and post score for their own skills in mentoring. [↑](#footnote-ref-2)
